# Evaluation of treadmill cardiopulmonary exercise testing and field measurement results in women’s youth and adult national team water polo players

**DOI:** 10.1016/j.heliyon.2024.e41131

**Published:** 2024-12-11

**Authors:** Mark Zamodics, Mate Babity, Attila Mihok, Csaba Bognar, Agnes Bucsko-Varga, Panka Kulcsar, Dora Boroncsok, Regina Benko, Alexandra Fabian, Balint Lakatos, Hajnalka Vago, Attila Kovacs, Bela Merkely, Orsolya Kiss

**Affiliations:** a Heart and Vascular Center, Semmelweis University, Budapest, 1122, Gaal Jozsef Street 9, Hungary; b Department of Sports Medicine, Semmelweis University, Budapest, 1122, Varosmajor Street 68, Hungary; c Gottsegen National Cardiovascular Center, Budapest, 1096, Haller Street 29, Hungary; d Hungarian Water Polo Federation, Budapest, 100, Alfred Hajos National Pool Complex, Hungary

**Keywords:** Athlete, Cardiopulmonary exercise testing, Field measurement, Physical fitness evaluation

## Abstract

The benefits of treadmill-based cardiopulmonary exercise testing (CPET) are well known. However, water polo trainings involve completely different movements in a distinct environment.

Our objective was to analyze data from elite youth and adult water polo players, gathered through CPET and age- and sport-specific swimming tests. Resting and exercise heart rate, as well as lactate levels, were examined at specific time points during both assessments. Additionally, maximal aerobic capacity was evaluated during the CPET.

Forty-six female water polo players were examined (age:18.5 ± 5.9 y, adults:19). No significant differences were found between CPET and swimming tests for resting heart rate (75[IQR:65–85] vs. 71[IQR:63–81] BPM, p = 0.33) and lactate levels (1.5[IQR:1.1–1.7] vs. 1.5[IQR:1.3–1.7] mM, p = 0.33). Maximal heart rates were higher during CPET than swimming (196.3 ± 9.7vs.191.0 ± 12.5 BPM, p < 0.001), while maximal lactate levels were lower (8.4 ± 2.4vs.9.6 ± 2.7 mM, p < 0.05).

Lactate levels remained elevated after routine cool-down swimming ordered by the trainers, but decreased after suggested further training (6.6 ± 2.7vs.3.7 ± 1.6 mM, p < 0.001). Comparing the youth (<18y) and the adult (≥18y) players, no differences in relative maximal aerobic capacity (44.4 ± 4.8vs.44.9 ± 5.5 ml/kg/min, p = 0.72) and in peak lactate values (8.2 ± 2.7vs.8.7 ± 1.9 mM, p = 0.48) were observed in CPET examinations, while maximal heart rates were higher in the youth group (200.7 ± 7.2vs.190.1 ± 9.6 BPM, p < 0.001).

Similarly, maximal lactate levels showed no differences (9.1 ± 2.6vs.10.4 ± 2.7 mM, p = 0.13), while maximal heart rates were higher in the youth group during swimming examinations (197.7 ± 10.0vs.181.4 ± 8.9 BPM, p < 0.001).

The combined use of both exercise tests enables the assessment of maximal physiological parameters (heart rate, lactate, aerobic capacity) during different types and intensities of physical exercise and identifies differences across age groups, facilitating the design of a more personalized and effective training program.

## Highlights

• The comparison of CPET and swimming tests provides an opportunity for a more accurate understanding of the two methods.
• Field measurements, though they have fewer opportunities than CPET, can reveal major training deficiencies, as our research showed.
• Comparing age groups highlights the youth team's shortcomings and age-group deviations.
• The combined use of the two methods promotes safe and successful elite sports.

## 1 Introduction

Recently, scientific background has become essential in elite sport activity. As the most obvious example, regular physiological assessments provide an opportunity for safer and more successful performance in sports. The possibilities of in-laboratory and field measurements are continuously broadening. However, the lack of objective scientific data may limit the usefulness of these examinations.

Cardiovascular screening extended with cardiopulmonary exercise testing (CPET) may also play role in the detection of cardiovascular and pulmonary diseases, in monitoring the progress of post-surgery and post-disease recovery, and in cardiology follow-up of athletes [1,2]. Moreover, the results of CPET are invaluable in legal performance enhancement and training planning [3,4]. Maximal intensity CPET measurements provide the opportunity to determine maximum heart rate, gas exchange, ventilatory and lactate thresholds, enabling the objective description of actual aerobic and anaerobic fitness and aiding the optimization of trainings. Due to the mixed nature of the physical activities during water polo trainings, determining aerobic and anaerobic fitness have great importance [5]. Although treadmill CPET is widely used in cardiology screening, we have little objective information about physiological maximal values of water athletes and about its well defined usefulness in physical fitness follow-up [6].

Sports cardiology studies do not only focus on clinical research but also evaluates athletes' performance through field measurements, during which athletes perform in their training environment, applying their sport-specific movements [7,8]. This is specifically important for water athletes, since these sport-specific movements completely differ from regularly applied CPET methods like running or cycling. By obtaining field measurements, our insight into the training loads during the athletes’ regular activity might be broadened, thereby a more individually optimized training planning could be achieved. The obvious disadvantage of these field assessments is that they are conducted outside of a controlled laboratory setting, which limits the number and the controllability of measurements that can be performed. Similarly to CPET results, there is a lack of objective values that can be applied for water polo athletes.

Moreover, data available in the literature about the relationship between the above two types of physical fitness assessments is particularly limited. It is also important to emphasize that physical fitness measurements of female athletes are remarkably underrepresented in the literature.

We also lack information about the physiological fitness parameters of different age groups of water athletes, and we particularly lack scientific data applicable for youth athletes. Comparing age groups allows us to understand differences between youth and adult groups, recognize deficiencies, and provide guidance for young athletes in training planning. Different protocols in the testing of different athlete age groups play an important role, as youth teams have fewer training hours and the dynamics of the matches also differ from those of adults.

Therefore, the goal of our research was to analyze and compare data from elite youth and adult female water polo players obtained through maximal intensity cardio-pulmonary exercise testing and field examinations. By scientifically and objectively comparing CPET and field measurements, we can gain new insights into the development of physical fitness parameters in different environments and during different physical activities of different age groups that may lead to a more comprehensive evaluation of elite athletes. Our data might assist both athletes and coaches in designing appropriate individual training programs, leading to a more successful and safer athletic performance.

## 2 Materials and methods

### 2.1 Study population

In our prospective study, a comprehensive examination was conducted on a cohort comprising 46 asymptomatic elite female water polo athletes, who were affiliated with either the national youth or adult teams and ranged in age from 13 to 31 years.

Youth players were defined as athletes under 18 years old, while adult players were defined as athletes ≥18 years old. Weekly training hours, height, and weight were significantly higher in adult players compared to youth athletes. (Table 1).

**Table 1:** Basic data of elite athletes participating in the study.

	Youth	Adult	Total
N	27	19	46
Age (years)	14.2 ± 0.5^a^	24.8 ± 4.0^a^	18.5 ± 5.9
Training (hours/week)	12.7 ± 3.0^a^	25.6 ± 6.3^a^	18.0 ± 7.9
Weight (kg)	62.0^a^ [IQR: 57.5–73.0]	74.5^a^ [IQR: 67.0–81.0]	67.5 [IQR: 61.0–75.5]
Height (cm)	168.0^a^ [IQR: 165.0–174.5]	178.0^a^ [IQR: 173.0–181.5]	172.5 [IQR: 167.3–178.8]

a *youth female vs. adult female p<0.001*.

A detailed questionnaire was implemented to record the data of sport activity as well as anamnestic medical data. Before laboratory CPET measurements, all athletes underwent physical examination, resting blood pressure measurement, and 12-lead resting ECG examination. All athletes included into the present study were free from injuries or significant health conditions (with particular regard to significant cardiovascular diseases, infections, metabolic diseases, psychological issues) that could have influenced the results.

Prior to the study, all participants and, in case of athletes <18 years old, all legal representatives gave written informed consent to the examinations. **The study was approved by the Medical Research Council of Hungary (Approval No.: IV/10282-1/2020/EKU) in compliance with the Ethical Guidelines of the Helsinki Declaration and Good Clinical Practice standards.**

A cardiology and sports medicine specialist supervised all examinations and data collection.

### 2.2 CPET examinations

**The maximal CPET measurements were performed on a treadmill ergometer, following a sport-specific incremental uphill-running protocol that has been long used and validated by our research team, with details available in our previous study** [3]. For inclusion in the study, it was mandatory to achieve maximal intensity at the CPET examination.

### 2.3 Field measurements

All examinations were performed at least 12 h after the previous training session. Age- and sport-specific maximal intensity freestyle swimming tests - representing training and match load - were developed by nationally qualified trainers and team physicians to reach the individually achievable maximal physiological values in the swimming pool. Following self-paced warm-up swimming of 400 m, the athletes were instructed to swim different distances due to age specific exercise capacity (youth: 2 × 400 m, adults: 1 × 400 m, 2 × 200 m, and 2 x 4 × 100 m) with achievable maximal intensity freestyle swimming. The athletes were instructed to swim as fast as possible during the full length of each swimming sessions. The time limit of the above periods of swimming was assigned, and after completing the swimming phases, the athletes spent the remaining time resting. At the end of the tests, two consecutive cool-down periods of 400-400 m long low-intensity self-paced swimming were assigned. All athletes were instructed to complete both cool-down sessions independently from the measured lactate values. The athletes were obliged to start the second cool-down swimming immediately after a lactate measurement carried out between the two sessions.

During the tests, continuous heart rate recording was carried out to collect resting, exercise, and recovery heart rate data by a system also validated for in-water measurements (Polar Team Pro, Kempele, Finland). Lactate levels were measured at defined time points of the swimming test: resting, between swimming periods, at the end of the last swimming phase, and following each cool-down periods (Lactate Scout 4+, EKF Diagnostik, Germany).

The time elapsed between the in-laboratory CPET and field tests was less than 1 month for both youth and adult athletes. The athletes were healthy throughout, and they were in the same training phase during both tests.

Due to the length and complexity of the tests, we did not align them with the athletes' menstrual cycles. Water polo athletes play matches every weekend, so athletes need to be able to perform at their maximum regardless of their menstrual cycle.

### 2.4 Statistical analyses

The data analysis for this paper was generated using the Real Statistics Resource Pack software (Release 8.9.1) (Copyright (2013–2023) Charles Zaiontz) and Microsoft Excel (Microsoft Corporation, USA) [11]. Descriptive statistics are presented as numbers (percentages), mean ± SD for normally distributed data, and median [interquartile range: 1st quartile – 3rd quartile (IQR: Q1–Q3)] for non-normally distributed data. The normality of the data was assessed using the Shapiro–Wilk test. The comprehensive statistical analysis' for dependent data were performed using either the paired Student's t-test or the Wilcoxon Signed Rank test, depending on data normality. For independent measurements, the unpaired Student’s t-test or the Wilcoxon rank-sum test (Mann–Whitney *U* test) was applied. Statistical significance was considered at p < 0.05. No missing data were present in the examined parameters for statistical analysis.

## 3 Results

### 3.1 Study population

#### 3.1.1 Comparisons between CPET and field measurements

**Fig. 1:**
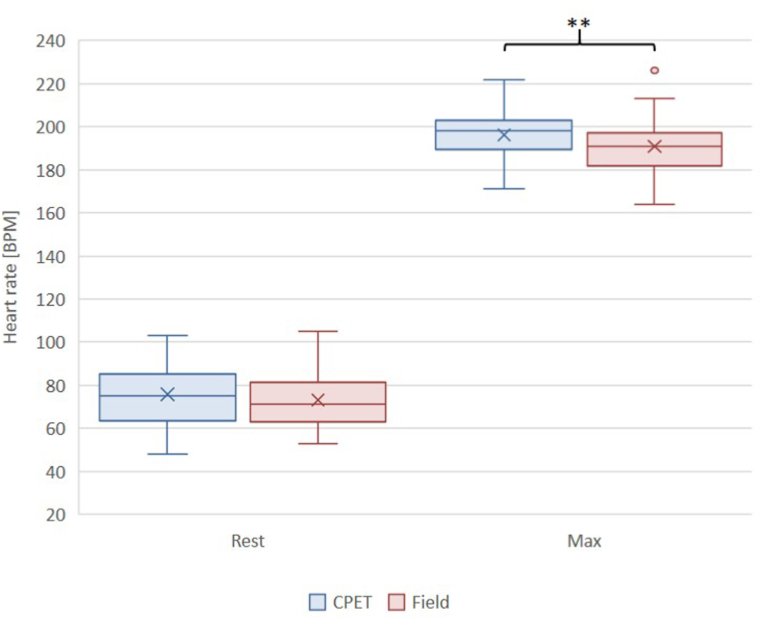
CPET vs. field measurement: comparison of resting and maximum heart rate values. Abbreviation: CPET: Cardiopulmonary exercise test; Field: Field measurement; Rest: Resting values; Max: Maximum values; ∗∗: p < 0,001.

**Fig. 2:**
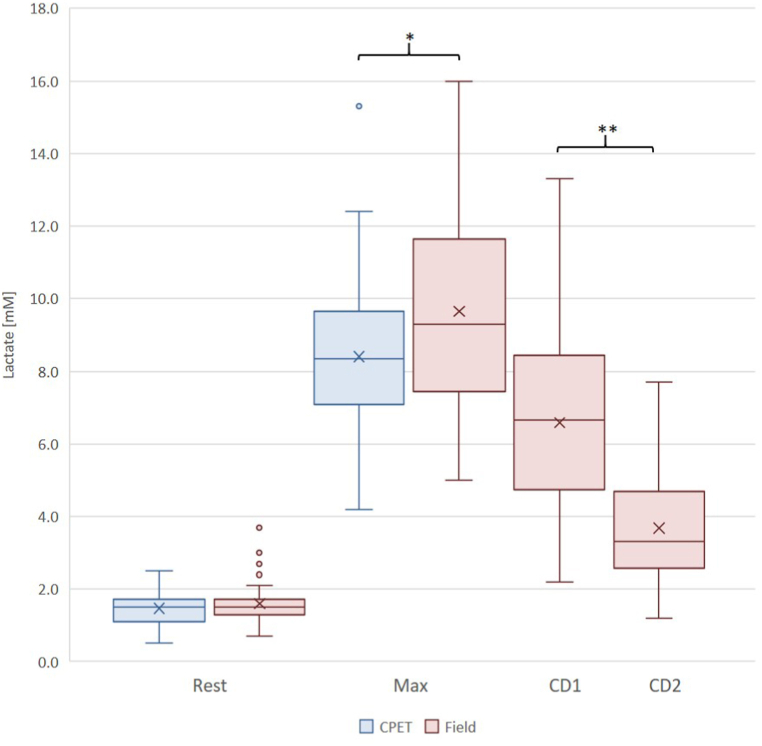
CPET vs. field measurement: comparison of resting, maximal and cool down lactate values. Abbreviation: CPET: Cardiopulmonary exercise test; Field: Field measurement; CD1-2: 1st and 2nd cool down swimming; Rest: Resting values; Max: Maximum values; ∗: p<0,05; ∗∗: p<0,001.

Comparing the basic resting parameters recorded before the CPET and field measurements, we could not find any differences between resting heart rate values (75 [IQR: 65–85] vs. 71 [IQR: 63–81] BPM, p = 0.33, Fig. 1.) and resting lactate levels (1.5 [IQR: 1.1–1.7] vs. 1.5 [IQR: 1.3–1.7] mM, Fig. 2.).

Maximal heart rates proved to be higher during CPET examinations than during field tests (196.3 ± 9.7 vs 191.0 ± 12.5 BPM, p < 0.001, Fig. 1.), while maximal lactate levels were lower during CPET examinations compared to field measurements (8.4 ± 2.4 vs 9.6 ± 2.7 mM, p < 0.05, Fig. 2.).

**Fig. 3:**
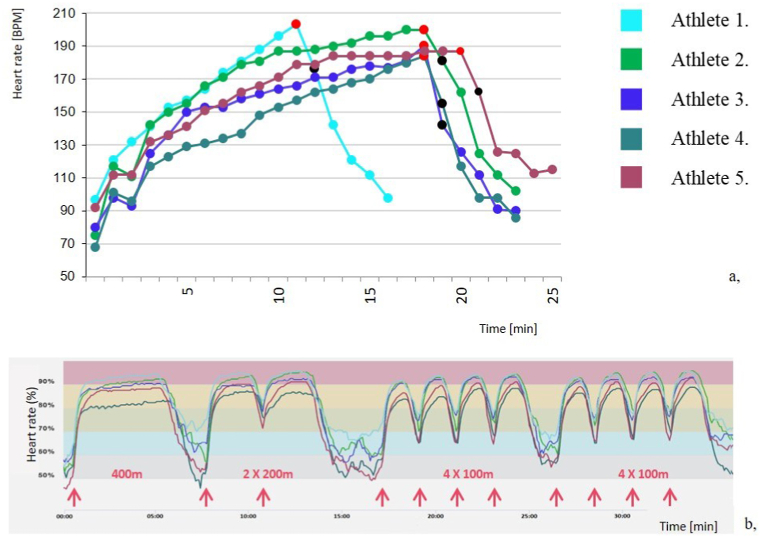
Continuous heart rate measurement results of 5 adult female water polo wing athletes a., during laboratory cardiopulmonary exercise testing and b., during the swimming tests.

Heart rate curves of CPET and field tests as a function of exercise duration for five female adult water polo wing players are shown in Fig. 3 [9]. Similarities between the two different testing methods can be clearly seen. For example, athlete 1, who had markedly higher heart rates during the running test, leading to a short exercise time, had similarly high heart rates during the maximal intensity swimming test compared to other athletes. Conversely, athlete 5, who had similarly short swimming times as athlete 1 referring to good exercise capacity, had an optimal, slowly increasing heart rate curve during the running test, also had lower heart rates during the swimming test. Similarities can also be observed between the kinetics of heart rate recovery during breaks in the swimming test, and at the recovery phase of CPET measurements. The heart rate recovery of athletes 1 and 2 is slower than that of athletes 3 and 4, as shown by the steepness of the slope of heart rate curves during both examinations.

Heart rate curves of individual athletes are denoted with the same colors on a. and b. figures. The red points represent the maximum heart rate, while the black points indicate 1-min post-exercise heart rate recovery. Heart rate zones denoted on Fig. 3/b were calculated from individual maximal heart rates determined during CPET examinations. Fig. 3a and b are reprinted from Sportorvostan by O. Kiss, H. Vago, B. Merkely, 2020, Semmelweis Kiadó. Reprinted with permission [9].

**Fig. 4:**
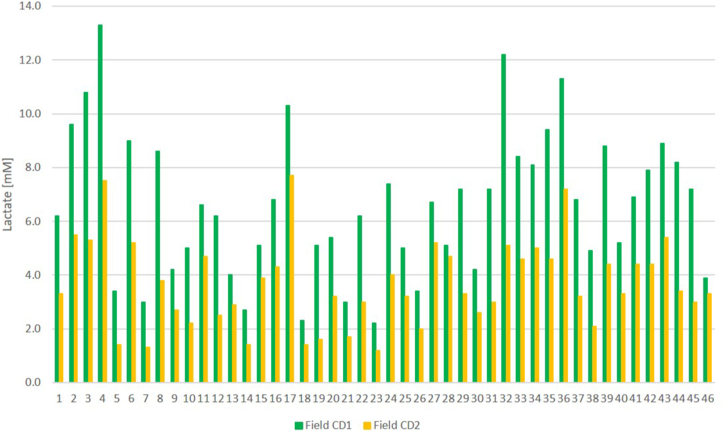
Individual lactate levels after the standard cool down swimming and after the additional second cool down swim. Abbreviation: Field CD1: field measurement 1st cold down swimming lactate values; Field CD2.: field measurement 2nd cool-down swimming lactate values.

After the standard cool-down swimming applied by the coaches, lactate levels remained remarkably elevated in all age groups, and in some individual athletes it still remained over 12 mM lactate levels (Fig. 2, Fig. 4). Therefore, we recommended additional cool-down swimming sessions, which further reduced the lactate levels in every group (youth: 6.0 ± 2.8 vs 3.4 ± 1.8 mM, p < 0.001, adult: 7.5 ± 2.2 vs 4.1 ± 1.2 mM, p < 0.001, summary: 6.6 ± 2.7 vs 3.7 ± 1.6 mM, p < 0.001, Fig. 2, Fig. 4.).

The detailed field measurement results of the groups are provided in the Supplement.

### 3.2 Comparisons between age groups

**Fig. 5:**
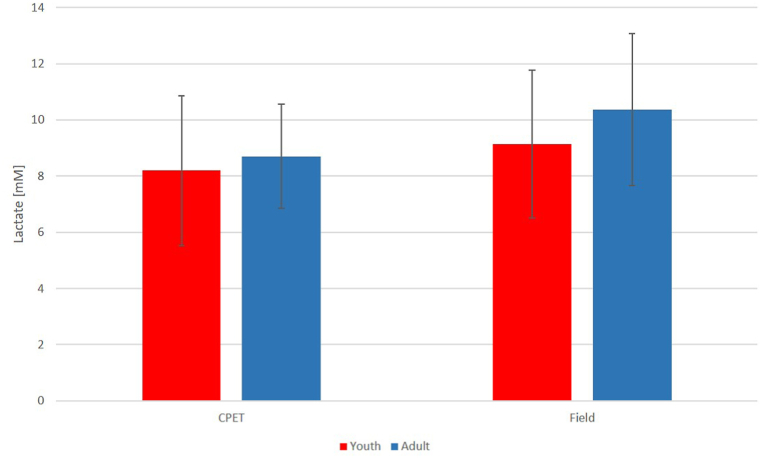
Comparison of age groups based on maximum lactate levels during maximal intensity CPET and swimming field tests. Abbreviations: CPET: cardiopulmonary exercise test, Field: field swimming test.

**Fig. 6:**
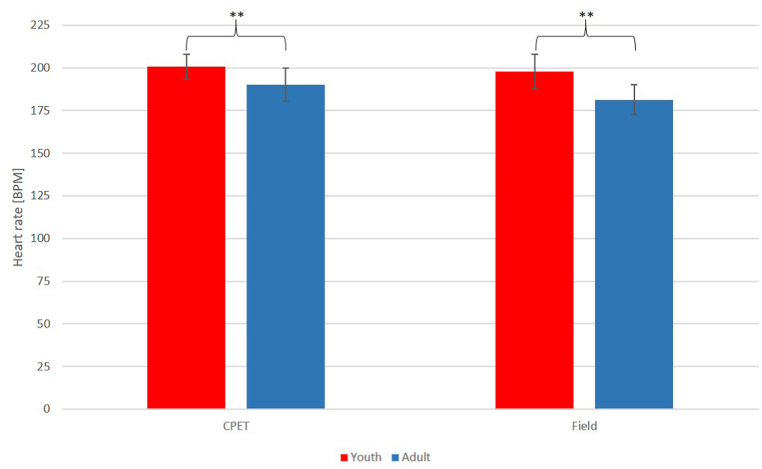
Comparison of age groups based on maximum heart rates during maximal intensity CPET and swimming field tests. Abbreviations: CPET: cardiopulmonary exercise test, Field: field swimming test, ∗∗: p<0.001.

When comparing the age groups, CPET examinations showed similar relative maximal aerobic capacity (44.4 ± 4.8 vs 44.9 ± 5.5 ml/kg/min, p = 0.72) and peak lactate values (8.2 ± 2.7 vs 8.7 ± 1.9 mM, p = 0.48, Fig. 5.). In terms of absolute oxygen uptake, youth players achieved significantly lower values (2.8 ± 0.3 vs 3.4 ± 0.5 l/min, p < 0.001) compared to adults. Maximum heart rates measured during CPET running were higher in the youth group (200.7 ± 7.2 vs 190.1 ± 9.6 BPM, p < 0.001, Fig. 6.) than in adults. However, there was no significant difference in maximum heart rate as a percentage of the age-predicted maximum (maximum heart rate/(220 - age) ∗ 100) between the two groups (97.0 ± 3.0 vs 97.4 ± 4.4 % p = 0.93).

Similarly to CPET, maximum lactate levels showed no differences (9.1 ± 2.6 vs 10.4 ± 2.7 mM, p = 0.13, Fig. 5.), while maximum heart rates were higher in the youth group (197.7 ± 10.0 vs 181.4 ± 8.9 BPM, p < 0.001, Fig. 6.) during field examinations. Additionally, during the field measurements, the youth athletes were able to achieve a higher maximum heart rate as a percentage of the age-predicted maximum (96.0 ± 5.0 vs 92.9 ± 3.7 %, p < 0.05) compared to the adult players."

## 4 Discussion

The main conclusion of our research is, that water polo players achieved higher heart rates and lower maximum lactate levels during CPET examinations compared to field measurements. When comparing age groups, it was observed that the younger group achieved higher heart rates in both examination methods than the adults, although the difference in maximum heart rate as a percentage of the age-predicted maximum was only observed during the field measurements. Moreover, no differences were seen in maximal lactate levels and relative maximal oxygen uptake.

As a starting point of our examinations, resting heart rate and resting lactate levels before the CPET and field tests showed no difference. However, resting heart rates were higher than expected in elite athletes. These results presumably refer to the similar psychological stress caused by either the swimming tests or the laboratory examinations. Accordingly, it can be stated that the athletes were in similar initial conditions during the two tests.

Individual heart rate curves hold essential information about the aerobic fitness of the athletes. If an athlete has an intensively increasing heart rate curve as compared to others following the same training protocol, such that it may limit exercise time, it indicates the need for individual training sessions to improve that athlete’s aerobic fitness. Conversely, if an athlete has an optimal, slowly increasing heart rate curve and a good heart rate recovery during exercise testing, this indicates good exercise capacity due to less energy demand and more optimal hemodynamic conditions of the circulatory system. Moreover, the follow-up of heart rate recovery as another parameter of anaerobic fitness provides information about how quickly the athlete is able to normalize physiological parameters in short resting phases; that is, an athlete with an optimal exercise heart rate curve is capable of tolerating longer exercise phases during a match situation with shorter resting phases needed between them. According to our results, the achieved maximal heart rates were higher during the treadmill CPET examination compared to the field measurements although both protocols involved maximal effort which required the maximum heart rate to be reached: CPET at the last phase of increasing intensity testing, while field measurements during all phases of swimming. In line with our results, a previous Brazilian study conducted by Alberton CL et al. examined 20 non-athlete females, who performed three different types of water exercises and underwent a treadmill exercise test as well [10]. According to the study, the maximum heart rate during the three different water exercises did not differ, although higher maximal heart rates were observed during the treadmill measurement. In another study of 14 coastguards, Meadly et al. were also able to observe higher maximal heart rates during the examination on treadmill as compared to swimming tests [11]. These results are consistent with the findings of our research. Previous research has proposed that the lower water temperature and the horizontal position of the body may play a role in this phenomenon [[12], [13], [14]]. In the modern world, when training alongside heart rate control, it matters which maximum heart rate value we use as a basis. Our research confirms that there are different physiological maximal heart rates during different types of training like CPET running and swimming sessions. However, many similarities could be found between heart rate kinetics of the athletes recorded during running and swimming tests.

Regarding lactate levels, it can be stated that the water polo athletes achieved higher lactate values during the swimming test than the running exercise. This result could refer to the fact that elite athletes may achieve higher lactate levels in their own types of physical exercise due to more intensive muscle work. It is also important to emphasize that our results may be influenced by differences between the applied exercise protocols. While intensive lactate increase occurs only in the anaerobic phase of the incremental CPET test, intensive muscle work also involving the arms is maintained throughout the whole length of the swimming tests causing intensive lactate elevation.

Besides the fact that maximal intensity swimmings require rather anaerobic exercise, the longer duration of swimming tests may also impact maximal lactate values, in addition to the involvement of more muscle groups. On the contrary, in the study conducted by Meadly et al., no significant difference in lactate values was found at maximum intensity between running and swimming tests however this result could be affected by the small sample size [11]. Due to the mixed nature of water polo, it involves a lot of anaerobic components both in training and competitions; therefore, the importance of lactate monitoring is remarkable [15,16]. The higher lactate values detected during the track measurements are noteworthy, as optimal lactate decrease also plays an essential role in recovery. The process of enhanced lactate clearance during low-intensity cool-down aerobic swimming involves the transfer of lactate from the muscles to the bloodstream, local blood circulation, and its uptake by the liver, skeletal muscles, and the heart. Lactate is partly converted into carbon dioxide and water, while it can also be a substrate of hepatic gluconeogenesis. During our swimming tests, coaches were forced to apply their standard cool-down swimming protocols, which were not sufficiently effective, as lactate levels remained high in most of the athletes. Therefore, additional swimming sets were required for proper cool-down [17]. These results emphasize the usefulness of field measurements. Differences between the ability of individual athletes to recover in the resting phases between physical load sessions do influence performance during competitions, and especially in team athletes like water polo players. Therefore, it is fundamental to record this parameter as a part of physical fitness follow-up, training planning and individually prescribed recovery strategy.

During the comparison of the age groups, we did not find any differences in terms of maximum relative VO2 values. The likely reason for this is the fact that in this age period, the muscle mass of an elite water polo athlete increases intensively due to the increasing training hours and hormonal changes during maturation. Therefore, although absolute VO2 max increases to maintain optimal cardiovascular adaptation, relative VO2 max (VO2 max divided by weight) will stay unchanged. On the whole, youth players achieved significantly lower values of absolute oxygen uptake as compared to adults. While previous research has described decreasing maximum VO2 values with advancing age, these studies typically focused on individuals aged 20 and older and did not consider the intensively improving youth/child age group [18,19]. In a study of young soccer players an increase in maximum relative VO2 was detected, but in this case, the participants were younger athletes between the ages of 8 and 18. In addition to physical development, the progressive increase in training hours may have also played a role in relative VO2 increase in this athlete group [20].

Similar to the maximum relative VO2 values, there was no difference among the age groups in the maximum lactate values during CPET. Similar to treadmill CPET measurements, we did not find any differences in terms of maximum lactate levels among the age groups during track measurements. The likely reason for this may be similar to that in the case of maximal aerobic capacity: as cardiovascular and metabolic adaptation follows intensive muscle mass increase, this may explain the lack of increasing maximal lactate levels with aging. In terms of maximum heart rate, the younger participants were able to achieve higher values in both testing methods. This is not immediately surprising, as the relationship between age and maximum heart rate is well known [21,22]; however, during the swimming tests, the difference between maximal heart rates was higher than it would have been estimated from age. This result may refer to the differences in exercise capacity between the age groups.

It is important to note that comparing two different exercise protocols can be challenging. Although there are relevant physiological differences between them, the aim of our study was to give practical information on comparing the two commonly used ways of testing elite water athletes: graded CPET with a swimming test designed to imitate match load. Our results assist sports professionals in interpreting CPET findings, thereby enhancing the collaboration between coaches and physicians, and contributing to the optimization of training.

Our results detailed above demonstrate the similarities but also the differences between two different physical fitness examinations in different age groups of female elite water polo players, indicating the need for the combined application of both, especially in the case of water athletes.

## 5 Conclusions

Understanding the interrelations of different forms of physical activity can yield substantial advantages not only in terms of performance and safety but also from a scientific perspective. Although field measurements and CPET results differ from several points of view, differences between age groups and the kinetics of the data are similar by our results. Cardiopulmonary exercise testing is indispensable for cardiovascular screening and the complex determination of physical fitness parameters, while field measurements provide an opportunity for determining sport-specific physiological maximum parameters during reproducible field tests planned by the trainers. Continuous heart rate as well as repeated lactate measurements are simple but informative ways of regular physical fitness measurements on the field. The kinetics of heart rate and lactate increase during maximal intensity field tests are either informative in team comparisons or in individual follow-up, and the measurements can be refined if heart rates are expressed in the % of CPET heart rates. Additionally, heart rate recovery and lactate measurements aid in planning appropriate post-exercise cool-down routines.

While ramp CPET testing provides information on both aerobic and anaerobic exercise capacity, maximal intensity field tests like the ones we applied in our study give information on a mostly anaerobic form of exercise. As water polo is a classical example of mixed team sports, information on both aerobic and anaerobic exercise capacity hold extremely important information.

The combined application and analysis of different exercise tests requiring enables safe and effective athletic performance and significantly contributes to the development of athletes. In particular, our research results contribute to a more effective use of treadmill CPET and field measurement data in water polo.

### 5.1 Limitations

In our research, only a relatively small number of heterogenous age elite female water polo players participated, so we cannot extrapolate the results to other age groups, males and athletes from other sports. This fact is particularly advantageous as well, since we have much less research available in the field of female athletes. We also plan to conduct the detailed examination of other age groups of female athletes, male water polo athletes and elite athletes from other sports as well. Another limiting factor is that we do not have a spirometry system applicable in water. Moreover, comparing measurement results of different exercise protocols is difficult to interpret and the analysis of the test results requires a complex approach.

## CRediT authorship contribution statement

**Mark Zamodics:** Writing – original draft, Investigation, Formal analysis, Conceptualization. **Mate Babity:** Writing – original draft, Investigation, Formal analysis, Conceptualization. **Attila Mihok:** Project administration, Formal analysis, Conceptualization. **Csaba Bognar:** Project administration, Investigation, Formal analysis. **Agnes Bucsko-Varga:** Project administration, Investigation, Formal analysis. **Panka Kulcsar:** Project administration, Investigation, Data curation. **Dora Boroncsok:** Project administration, Investigation, Formal analysis. **Regina Benko:** Project administration, Investigation, Formal analysis. **Alexandra Fabian:** Project administration, Investigation, Formal analysis. **Balint Lakatos:** Project administration, Investigation, Formal analysis. **Hajnalka Vago:** Project administration, Investigation, Formal analysis. **Attila Kovacs:** Project administration, Investigation, Formal analysis. **Bela Merkely:** Writing – review & editing, Supervision, Investigation, Funding acquisition, Data curation, Conceptualization. **Orsolya Kiss:** Writing – review & editing, Supervision, Investigation, Funding acquisition, Data curation, Conceptualization.

## Ethics approval

Prior to the study, all participants and, in case of athletes <18 years old, all legal representatives gave written informed consent to the examinations. **The study was approved by the Medical Research Council of Hungary (Approval No.: IV/10282-1/2020/EKU) in compliance with the Ethical Guidelines of the Helsinki Declaration and Good Clinical Practice standards.**

The present study complies with the current laws of the country in which it was performed.

## Availability of data and material

The datasets generated and analyzed during the current study are not publicly available, but are available from the corresponding author on reasonable request.

## Funding

Support by the 10.13039/501100000780European Union project RRF-2.3.1-21-2022-00004 within the framework of the 10.13039/100020626Artificial Intelligence National Laboratory. This project was supported by a grant from the 10.13039/501100018818National Research, Development and Innovation Office (10.13039/501100011019NKFIH) of Hungary (K 135076).

Project no. TKP2021-NKTA-46 has been implemented with the support provided by the 10.13039/501100024593Ministry of Innovation and Technology of Hungary from the National Research, Development and Innovation Fund, financed under the TKP2021-NKTA funding scheme. MZ and MB: Development of scientific workshops of medical, health sciences and pharmaceutical educations (EFOP-3.6.3-VEKOP-16-2017-00009). MB: Supported by the ÚNKP-23-3 New National Excellence Program of the 10.13039/501100015498Ministry for Innovation and Technology from the Source of the National Research, Development and Innovation fund.

The funding sources had no involvement in study design, in the collection, analysis and interpretation of data, in writing the report and in the decision to submit the article for publication.

## Declaration of competing interest

The authors declare that they have no known competing financial interests or personal relationships that could have appeared to influence the work reported in this paper.
